# Literary Notes

**Published:** 1899-05

**Authors:** 


### *LITERARY NOTES.*

### Gerrish's Anatomy by American Authors.

Gerrish's forthcoming "Anatomy by American Authors promises to be the work for which teachers and students have long been looking. Its editor, Professor S. H. Gerrish of Portland, has selected as his fellow-contributors leading anatomists throughout the country, wisely restricting their number to accord with the best division of the subject, gaining thereby unity in result joined with the highest authority. The list includes Professors Bevan of Rush in Chicago, Keiller of the University of Texas, McMurrich of the University of Michigan, Stewart of the University-Bellevue College in New York, Woolsey of Cornell Medical College likewise in New York, and Gerrish himself, who is not only editor but perhaps the largest contributor.

The plan of the work judiciously avoids the unimportant and exceptional, reserving its space for those portions of anatomical knowledge which are necessary to the intelligent study of physiology, surgery and internal medicine. The authors have endeavored to stand in the place of a living teacher to the student, selecting such portions as will be of actual service to the pupil in his study and to the practitioner in his subsequent clinical work, clarifying obscurities, giving most help in the most difficult parts, and illustrating everything by all available methods. Pictorially "Gerrish's Anatomy" will be by far the most lavish work ever offered on a subject which can already boast of many elaborately illustrated text-books. The engravings number about one thousand, their size is large enough to make visible every detail, colors have been employed more liberally than ever before, and lastly the labels of the parts have been conspicuously engraved upon them whereby a glance gives not only their names but also their position, extent and relations, obviating entirely the slow, toilsome and wasteful mental processes necessitated where only reference letters are employed.

In an early issue we shall give our readers a review of the book itself.

### MAY LADIES' HOME JOURNAL.

"The Countess Emilia," Anthony Hope's new romance, is begun in the May *Ladies' Home Journal*, and "The Art of Listening to a Sermon" inaugurates the first of a series of articles on the pulpit and the pew by Ian Maclaren. Another notable feature of the same issue is "The Secrets of a Happy Life," by the Rev. Newell Dwight Hillis, D.D., pastor of Plymouth Church, Brooklyn, who has become a regular contributor to the *Journal*. Paul Leicester Ford writes "The Anecdotal Side of George Washington," recounting some of the best but least-known stories of the "Father of His Country." Viola Allen draws upon her own rich stores of experience to tell "What it Means to be an Actress," and Joseph Edgar Chamberlin introduces "Helen Keller as She Really Is," giving some interesting glimpses of this marvelous blind and deaf girl.

On the editorial page Edward Bok treats of the pretty American girls, and discourses on the most-beloved women of the century. The feminine wardrobe is considered in elaborate detail, the articles being by the best fashion writers—and illustrated. "The Building of the Ship" is the theme of the sixth of W. L. Taylor's series of illustrations of Longfellow's poems, and pictorial features of practical interest are "Nature's Garden," "The Prettiest Country Homes in America," Rustic Arbors and Summer Houses" and "The Flag in the Church." Maria Parloa inaugurates a new department, "Household Helps and New Ideas," and Mrs. S. T. Rorer gives the menus of "Little Dinners by Eighteen of My Girls," and writes of "Milk: Its Use and Abuse." Helen Watterson Moody defines "The True Meaning of Motherhood," and Mrs. Humphry contributes her second article on "How to be Pretty Though Plain." In short, the May *Journal* has apparently anticipated every need that can arise in the home. By the Curtis Publishing Company, Philadelphia. One dollar per year; ten cents per copy.

---

WE are in receipt of a beautiful lithographed pamphlet from the ever enterprising Mr. Henry of Louisville, who in a letter informs us of his now being the principal owner of the famous French Lick Springs of Indiana, capacity five hundred guests. The celebrated Spa "Pluto" Americas Aperient he will introduce at once, through the *Medical Press*, as the most saline hydragogue eliminant and intestinal antiseptic akin to Carlsbad, without the accompanying nausea and thirst.

